# MicroRNA Target Identification—Experimental Approaches

**DOI:** 10.3390/biology2010189

**Published:** 2013-01-25

**Authors:** Aida Martinez-Sanchez, Chris L. Murphy

**Affiliations:** Kennedy Institute of Rheumatology, Nuffield Department of Orthopaedics, Rheumatology and Musculoskeletal Sciences, University of Oxford, 65 Aspenlea Road, London W6 8LH, UK; E-Mail: aida.martinez-sanchez@kennedy.ox.ac.uk

**Keywords:** miRNA, gene expression, miRISC, miRNA target identification, RIP-Seq, HITS-CLIP, HT-Seq

## Abstract

MicroRNAs (miRNAs) are small non-coding RNA molecules of 21–23 nucleotides that control gene expression at the post-transcriptional level. They have been shown to play a vital role in a wide variety of biological processes and dysregulated expression of miRNAs is observed in many pathologies. Understanding the mechanism of action and identifying functionally important mRNA targets of a specific miRNA are essential to unravelling its biological function and to assist miRNA-based drug development. This review summarizes the current understanding of the mechanistic aspects of miRNA-mediated gene repression and focuses on the different approaches for miRNA target identification that have been proposed in recent years.

## 1. Introduction

MiRNAs are a large family of ~22 nucleotide RNAs which regulate virtually every aspect of biology, including development, proliferation, differentiation and metabolism [1]. It is therefore not surprising that disruption of miRNA function contributes to many human diseases, including cardiovascular disorders, cancer and complex genetic diseases [2,3,4].

MiRNAs are processed from precursor molecules (pri-miRNAs) which are normally transcribed by RNA polymerase II [5]. In animals, the majority (80%) of miRNA genes are located in introns of both protein-coding and non-coding genes [6]. Thus, expression of a large subset of mammalian miRNAs may be transcriptionally linked to the expression of other genes, allowing for co-ordinate regulation of miRNA and protein expression [7]. The pri-miRNA is processed into the mature miRNA in a two-step manner. Briefly, in the nucleous, the RNAse III enzyme DROSHA, in complex with other proteins such as DGCR8 in mammals, cleaves the pri-miRNA into a ~70 nucleotide precursor (pre-miRNA) which is transported to the cytoplasm by exportin 5. Once in the cytoplasm, the pre-miRNA is further processed by another RNAse III enzyme—DICER, acting in conjunction with the RNA binding protein TRBP in mammals. As a result of this second cleavage a small RNA duplex (~20 nucleotides) is generated. In general, one of the strands (known as the guide) is incorporated into a miRNA-induced silencing complex (miRISC) while the other one (passenger strand) is released and degraded [8]. Further studies have confirmed that, in some cases, the passenger strand can also be loaded into miRISC, thus also functioning as a mature miRNA [9].

The core of miRISC is formed by the Argonaute (Ago) proteins. Different members of this family have specific expression patterns, binding partners and biochemical capabilities [10]. Indeed only Ago2 (out of the four Ago proteins expressed in humans) is capable of mediating endonucleolytic cleavage of the target mRNA which occurs when there is complete complementarity between a miRNA and a target site [11]. Nevertheless, most metazoan miRNAs direct RISC to their target mRNAs by interacting with sites of imperfect complementarity. As a result, the miRNA promotes the degradation and/or inhibits the translation of the target mRNA, resulting in repression of its expression [12].

In general, the most important region for target recognition comprises nucleotides 2–8 of the miRNA (known as the “seed” region); and binding sites are most commonly found in the 3' UTR of the cognate mRNAs [13]. Despite this, many examples have emerged recently showing that miRNA binding sites can be located outside the 3' UTR (commonly in the coding region), and even lack of perfect seed pairing can sometimes be compensated by 3' complementarity or centered pairing [14,15,16]. Thus, predicting whether a mRNA will be regulated by a given miRNA in the endogenous context is challenging and gets more complicated still when taking into account the different factors that can also control the ability of a miRISC to bind and repress specific targets [17,18].

During the last decade, several bioinformatic tools have been developed to predict miRNA targets [19]. Algorithms are predominantly based on seed pairing and evolutionary conservation, typically predicting hundreds, even thousands of targets for each miRNA, including a high proportion of false-positives and false-negatives [20].

The difficulty and complexity of identifying endogenous miRNA targets has resulted in the generation of a wide variety of experimental approaches, from identification of mRNAs/proteins regulated by overexpressing or antagonizing a given miRNA to genome-wide approaches including immunoprecipitation of miRISC [21]. In this review, we first briefly present the molecular mechanisms that drive miRNA silencing. Next we summarize the bioinformatic tools currently available for miRNA target prediction and finally we discuss in detail the different experimental approaches that have been undertaken in recent years, paying special attention to their potential limitations for identifying biologically relevant targets.

## 2. Mechanisms of miRNA Repression

The exact mechanisms used by miRNAs to regulate gene expression remain unclear and have been a controversial subject in recent years. Evidence for translational repression, mRNA destabilization and even activation of gene expression has been reported [22].

Initial work in animals pointed to regulation at the level of mRNA translation. Translation in mammals requires numerous factors that allow the recruitment of the ribosomal subunits to the mRNA initiation codon (initiation), the elongation of the nascent polypeptide chain (elongation) and the release of the mature protein (termination). Some of these factors recognize the 5' Cap or the 3' UTR tail of the mature mRNAs allowing its efficient translation [23]. Although most of the studies pointed to miRNAs as inhibitors of mRNA translation initiation [24,25], inhibition of elongation, premature termination of translation and co-translational protein degradation have also been described [22,26,27,28,29].

Whereas perfect pairing of an entire miRNA with its target resulting in endonucleolytic cleavage is a rare mechanism in animals, exonucleolytic degradation often occurs. Thus miRNAs promote the recruitment of deadenylation factors to the target mRNA which remove its poly(A) tail making the mRNA more susceptible to exonucleolytic degradation. Indeed, recent work shows that binding of some of these factors, such as CCR4-NOT promotes deadenylation to repress translational initiation [30,31].

Several groups have taken advantage of advances in mass spectrometry and RNA sequencing to study, on a genome-wide scale, the effects of miRNA action on mRNA and protein levels after miRNA ectopic expression or removal in animals [32,33]. Although, in general, these studies conclude that degradation of miRNA targets is a widespread effect of miRNA action and provides a major contribution to silencing by animal miRNAs [34], whether target degradation occurs as a consequence of an initial block in translation remains an unresolved question. The fact that some authors have found hundreds of miRNA targets for which protein levels were decreased far more than mRNA levels [35] supports the idea that the precise mechanism of action is highly cell type and/or target-specific dependant. The effects of miRNAs on both plant and animal mRNA translation and decay have been comprehensively reviewed elsewhere [12,22,36].

## 3. Computational miRNA Target Identification

The best characterized features determining miRNA-target recognition are six-nucleotide (nt) seed binding sites, which perfectly complement the 5' end of the miRNA (positions 2–7) [13]. Also, a match with miRNA nucleotide 8, an A across from nucleotide 1, or both of these conditions, augment the seed pairing and enforce miRNA-mediated repression [37]. These seed-pairing “rules” are widely used to predict functional miRNA target-sites, normally in combination with the secondary structure of the 3' UTR, the neighbouring contextual information and/or evolutionary conservation [13,38,39]. It has been recently shown that miRNAs can also effectively repress the expression of their targets through “G-bulged sites”, which include a G-bulge in position 5–6 of the seed-complementary region [40]. Based on that, in recent years several miRNA target prediction programs have been published [20,41] with characteristic features summarized in Table 1.

**Table 1:** Characteristic features of the most commonly used programs for prediction of miRNA targets.

Program	Reference	Detection capacity	Seed-match	Allows mismatches	Sequence analyzed in target	Thermodynamics and/or secondary structure of the duplex	Conservation	Considers non-conservative sites	Additional features
DIANA-microT	[42]	mRNA targets for a given miRNA; miRNAs targeting a given mRNA; Specific miRNA/mRNA interactions	7-9 nt long	Additional 3' pairing; single G:U wobble pair; 6 nt seed	3' UTR	+	+	+	Signal to noise ratio and precision score for evaluation of significance
DIANA-microT-CDS	[43]	mRNA targets for a given miRNA; miRNAs targeting a given mRNA; Specific miRNA/mRNA interactions	7-9 nt long	Additional 3' pairing; single G:U wobble pair; 6 nt seed	3' UTR + CDS	+	+	+	Signal to noise ratio and precision score for evaluation of significance
microInspector	[44]	miRNAs targetting a given mRNA; Specific miRNA/mRNA interactions	6 nt long	4-5 nt seed with additional G:U pair	ANY (submitted by the user)	+			Allows identification of weak interactions in any reagion of the mRNA
miRanda	[45]	mRNA targets for a given miRNA; miRNAs targeting a given mRNA; Specific miRNA/mRNA interactions	Positions 2-8 given higher weigth	Some mismatches allowed	3' UTR	+	+ (PhastCons score)	+	MicroRNA expression profile available. mirSVR score available
Pictar	[46]	mRNA targets for a given miRNA; miRNAs targetting a given mRNA	7 nt	Single G:U wobble pair allowed	3' UTR	+	+		Accounts for synergistic effects of several miRNAs/same miRNA binding
RNA22	[47]	mRNA targets for a given miRNA; miRNAs targeting a given mRNA; Specific miRNA/mRNA interactions	6-7 nt	Optional 1-2 mismatches	ANY (submitted by the user)	+			Permits identification of binding sites even if the miRNA is unknown
RNAhybrid	[48]	Specific miRNA/mRNA interactions	Optional	Optional	ANY (submitted by the user)	+			Variation of secondary structure prediction, seed is given little weigth
TargetScan (v6)	[49]	mRNA targets for a given miRNA; miRNAs targeting a given mRNA; Specific miRNA/mRNA interactions	6-7 nt (+ A in position 1)	Allowed if compensation by conserved 3' pairing	3' UTR	+	+	+ (very low scores)	Importance of the context score: local AU content, position in the 3' UTR, site abundance.

Additional information resources record nomenclature, sequence annotation such as genomic organization, precursor sequences, literature citations and links to target prediction sites (miRBase [50]; miRGen [51]), experimentally validated microRNA targets (TarBase [52]) or microRNA expression profiles (miRNAMap [53]; smiRNAdb).

The fact that animal miRNAs have only limited complementarity to their target sites means that even small differences in algorithms used produce a big difference in target predictions. Although the common assumption that targets predicted by more than one program are more accurate, this does not appear to be the case. A few recent papers have performed very interesting studies where miRNA binding sites predicted by different algorithms were tested against genes proposed as targets by experimental evidence [20,54]. The major conclusion of such studies was that, in general, programs that rely on evolutionary conservation of the mRNA sequence complementary to the seed tend to have relatively high precision but low sensitivity. The same occurs when a combination of algorithms is used: better specificity is achieved at the cost of reduced sensitivity. In practice, this yields a very good performance of these bioinformatics tools for prediction of highly conserved consensus binding sites (an accuracy of ~50% in many cases [20]) but a very low efficiency for the detection of non-conserved binding sites, as well as for those binding sites with poor pairing in the seed sequence. Similarly, one of the biggest limitations of current algorithms is that the predictions are in most cases restricted to the 3' UTRs, when recent experimental data indicate that a large proportion of the miRNA/mRNA interactions may occur through the coding sequences (CDS) or even the 5' UTR [16]. In addition, the binding rules for miRNA/target interactions through the 3' UTR could be different than through other mRNA regions, further reducing the capacity of current logarithms to predict these interactions.

Finally, none of these methods takes into account the possibility of tissue-specific interactions. Thus, experimental approaches for miRNA target determination are not only indispensable to confirm the biologically relevant targets of a given miRNA but are also essential to uncover further rules (both general and specific) of miRNA target binding. In this way, experimentally-based (and validated) data will feed back to significantly improve and refine the performance of computational predictions of miRNA-target interactions.

## 4. Experimental miRNA Target Identification

Although many miRNAs and their binding sites are highly conserved, suggesting an important function, a typical miRNA-target interaction produces only a subtle reduction (< 2 fold) in protein level of its target and many miRNAs can be inhibited or deleted without generating any obvious phenotype. Accordingly, an emerging view is that miRNAs may act to confer robustness to biological processes, for example, by reinforcing transcriptional programs to sharpen developmental transitions and entrench cellular identities or buffering fluctuations in gene expression, sharpening the cell response to stress signals or certain regulatory networks [55]. This does not make it any easier to identify the relevant targets of a given miRNA. Indeed, when bioinformatics tools have been used to pre-select the putative targets of a given miRNA, the vast number of predicted targets (usually with very disparate functions and including a high percentage of false-positives and false-negatives) challenges scientists to choose which ones are worthy to validate experimentally, and which ones will have a major impact in a given biological process under study. Although methods for target validation will also be discussed in this section, we will first focus on those experimental approaches that allow the researcher to directly determine the actual targets in a specific biological system, avoiding the biases inherent in the use of computational predictions.

High-throughput methods for identifying miRNA targets have been developed using a wide variety of experimental technologies, normally in combination with overexpression/inhibition of the miRNA, and in some cases with biochemical isolation of target mRNAs that are bound to miRNAs [56].

### 4.1. Expression Profiling Following miRNA Overexpression/Inhibition

Since microRNAs act by inhibiting translation and/or promoting degradation of their targets, the most straight-forward approaches rely on transfection of specific miRNA mimics or inhibitors into the cells followed by high-throughput analysis of mRNA expression (by microarray or high-throughput sequencing (HT-Seq), or proteomics [57].

Initial studies transiently transfected miR-1 (muscle-specific) and miR-124a (brain-specific) into Hela cells, where they are not normally expressed, and used microarray analysis to identify those mRNAs downregulated as a consequence [58]. Similar studies where soon performed [37,59] showing that mRNA transcripts downregulated after miRNA overexpression were significantly enriched for matches to the seed sequence. To avoid the bias that could be caused by the delivery of supraphysiological levels of an exogenous miRNA, the same kind of approach has been applied using antisense oligonucleotides that inhibit miRNA action [60], observing, for example, that a high number of transcripts were increased upon inhibition of miR-122 in the liver [61]. Despite the often beautiful work presented by these groups, introduction of miRNA inhibitors in primary cells or tissues is challenging (as is the inhibition of an endogenously highly expressed miRNA), and therefore most of the results have been obtained by overexpression of miRNAs in cell lines. Interestingly, when inhibition of miR-15 and miR-16 was performed in addition to overexpression experiments [59], the increase in mRNA expression after inhibition was small compared to the downregulation observed after overexpression. Thus, the effect of ectopic miRNAs might be easier to detect and therefore a more useful tool, although potentially more prone to artifacts. Technical advances in next-generation sequencing technology have now enabled the use of RNA-Seq as an alternative to microarray gene expression analysis, providing a larger list of inferred miRNA targets in over-expression studies [62]. The higher sensitivity of this technology may be especially useful when miRNA inhibition is performed, since small changes in RNA levels can be more reliably detected.

The transcriptome profiling approach has two clear limitations: first, this method cannot distinguish direct from indirect targets (which can be partially minimized by harvesting the cells soon after transfection) and second, those targets that are only regulated at the level of translation, occurring without significant alteration of the transcript levels, will not be detected. This second handicap can be addressed by the use of proteomics. Proteomic approaches have the inherent advantage of assaying the ultimate effect of miRNAs. Newly synthetized proteins can be metabolically labelled by growing cells in medium containing heavy isotopes of essential amino acids (SILAC: Stable isotope labelling by amino acids in cell culture). Mass spectrometry can then be used to determine the ratio of peptide peak intensities from the light and heavy isotopes as a measurement of protein synthesis and the differences can be assessed after overexpression/inhibition of a given miRNA [35,63]. This approach was successfully used, for example, to determine targets of (overexpressed) miR-1, miR-124 and miR-181 in Hela cells and of miR-223 using mouse neutrophils isolated from miR-233 deficient mice [32], finding that, for most of the targets changes in mRNA transcript levels were also detected. A similar correlation has been detected in several other studies, as well as an enrichment in seed matching sequences in the detected targets [35,64].

### 4.2. Polysome Profiling Following miRNA Overexpression/Inhibition

A mRNA that is being actively translated will be bound to a high density of ribosomes. Accordingly, a ribosome-profiling strategy is based on the recovery of the mRNA fragments that are bound by ribosomes (and thus protected from the action of RNAses), and identification of those fragments by deep sequencing. The abundance of different fragments corresponding to an mRNA is a direct indication of the amount of translation of that gene. [65]. This approach was used in 2010 by Bartel’s group in combination with overexpression of miR-1 or miR-155 in HeLa cells, which do not normally express these miRNAs [34]. They transfected HeLa cells with miR-155/miR-1 mimics and treated them with cycloheximide to arrest the translating ribosomes. Next, they digested the non-protected RNA with RNAseI and purified the resultant monosomes by a sucrose gradient. Those monosomes contained RNA fragments of ~30 nucleotides, which were released and identified by high-throughput sequencing (Ribosomal protected RNA fragments, or RPF). In parallel, total mRNA after miRNA overexpression was also sequenced to assess the contribution of mRNA stability (RNA-Seq) *versus* translational repression (RPF) with regard to the miRNA mechanism of action. The same experiment was performed with neutrophils obtained from a miR-223 knockout mouse *versus* wild-type, which express high levels of miR-223. They found that expression of genes with at least one predicted miR-155 or miR-1 binding site in their 3' UTR was lower after miR-155 or miR-1 transfection in both RNA-Seq and RPF samples. Conversely, genes with at least one binding site for miR-223 were over-represented in RNA-Seq and RPF from miR-223 null neutrophils. By using this approach, the main finding of these authors was that at least 84% of the miRNA-mediated repression was due to mRNA destabilization, since only minor differences were found between the RPF (measure of translation) and the RNA-Seq (measure of RNA degradation) after miRNA modulation. Nevertheless, using a similar technique, Bazzani and co-workers [66] found that the effects of miR-430 in zebrafish occur at the level of translation preceding RNA decay, so this disparity may result from the steady-state conditions used, the biological system, or the miRNA under study.

In general, it has been assumed that changes in mRNA levels closely reflect the impact of miRNAs on gene expression [34] and, given that proteomics approaches are more expensive, less sensitive and technically more complex than mRNA profiling techniques, it is not surprising that most of the publications generated are based on transcriptome analysis. Ribosomal profiling, on the other hand, is technically very challenging, and although a recently developed technique, the quick reduction in the cost of the HT-Seq makes it probable that this approach will be increasingly used in the future in combination with mRNA profiling.

Nevertheless, importantly, none of these techniques allows distinction between direct and indirect targets, and, in addition to altered expression, candidate targets are normally selected based on (imperfect) computational predictions (as previously discussed). Therefore, during the last few years efforts have been directed to the development of unbiased techniques to enable efficient and unambiguous determination of direct miRNA targets, *i.e.*, those targets found in the cell specifically in association with the miRNA effector complex, *i.e.*, miRISC.

### 4.3. Pull-Down Assays with Members of miRISC

The mammalian miRISC is the means by which mature miRNAs bind their mRNA targets, and they contain several proteins, including an AGO protein and GW182 [67,68]. Different methods have been developed to recover those mRNAs bound by miRISC (and therefore direct miRNA targets) using pulldown or immunoprecipitation of a component of miRISC (both a native member or an epitope-tagged one), normally in combination with overexpression/inhibition of the miRNA of interest. The recovered mRNAs can then be detected individually by RT-qPCR, or more commonly by high-throughput techniques such as microarrays or HT-Seq.

#### 4.3.1. Tagged miRNA Pull-Down

Ørom and colleagues developed a direct affinity purification method for experimental identification of miRNA targets based on the transfection into cells of synthetic miRNA duplexes carrying a biotin group attached to the 3' end of the miRNA sense strand. These sense strands are incorporated into miRISC and, after cell lysis, the miRNA-mRNA complexes are captured on streptavidin beads from which the mRNA species can be purified and analysed [56]. This technique led to the discovery of miR-10a interaction with the 5' UTR of ribosomal protein transcripts which surprisingly enhanced their translation and independently demonstrated previously detected targets of miRNAs *bantam* and miR-124 in cell lines [69]. Nevertheless this study is rather controversial for several reasons: firstly, the pulled down mRNAs were not enriched for miR-10a seed matches; secondly, the fact that they were mostly abundant ribosomal mRNAs suggest they might have associated with the biotinylated mRNA non-specifically (it is not known what effect the biotin tag may have on miRNA binding); and finally, as already mentioned, most identified genes were translationally upregulated, rather than downregulated, which the authors attribute to the presence of binding sites in the 5' UTRs. Thus, the ability of this technique to comprehensively identify true miRNA targets has yet to be fully validated.

An *in vitro* variation of this technique called LAMP (Labeled microRNA pull-down assay) utilizes digoxigenin (DIG)-labelled pre-miRNA oligonocleotides that are mixed with cell extracts. Labelled extracts are immunoprecipitated with anti-DIG antibodies before analysis of the co-immunoprecipitated mRNAs [70].

A recently developed alternative to biotin-labelling is so-called miR-TRAP (miRNA target affinity purification) in which the miRNA is conjugated to psoralen to produce a highly photo-reactive probe. The labelled miRNAs function similarly to endogenous miRNAs, and when the cells are exposed to UVA radiation (360 nM which is less harmful than the 254 nM used in other crosslinking experiments—a consideration relevant for *in vivo* experiments) the Pso moiety of the miRNA reacts with uridin on target mRNAs, enabling the bound complex to be stringently purified by biotin-streptavidin affinity purification. The biotin is incorporated in the 3' UTR of the miRNA as an affinity tag [71]. The authors have successfully used this approach to detect two novel targets of miR-15b and are currently applying these methods to assess miRNA targets in various disease models. Although susceptible to the same handicaps as the more simply labelled biotinylated miRNA based technique, the covalent link between the Psoralen-tagged miRNAs and target mRNAs allows the use of much more stringent purification conditions, which may diminish the recovery of non-specific targets.

Interestingly, all of these methods could be modified to identify miRNAs targeting a mRNA of interest by replacing labelled miRNA with labelled transcript. Along these lines Yoon and colleagues proposed a systematic approach termed MS2-TRAP (tagged RNA affinity purification) for identifying miRNAs associated with a target transcript in a cellular context. Briefly, they tagged the mouse linRNA-p21 with MS2 hairpins and co-expressed it in mouse embryonic fibroblasts (MEFs) along with the chimeric protein MS2-GST. They then affinity-purified the miRNAs present in the RNP complexes using glutathione-SH beads and finally measured them by qPCR. Out of the 5 miRNAs analysed (predicted to target linRNA-p21), 4 were enriched in the pulldown and two were functionally validated [72]. This approach could be widely used if coupling of the pulldown to high-throughput approaches for miRNA detection were performed. Nevertheless, one of its biggest limitations is that both the tagged RNA and the MS2-protein have to be exogenously introduced, which restricts its use to cell lines since there is typically low efficiency of transfection of such reagents in primary cells or in an *in vivo* setting.

#### 4.3.2. Immunoprecipitation of miRISC Proteins

Regardless of subsequent mechanism of action, all miRNAs first bind to the target mRNAs in the miRISC. Different approaches have been developed in order to recover and detect those mRNAs co-immunoprecipitated with RISC proteins. To identify targets of specific miRNAs, cells are transfected with a synthetic miRNA or a miRNA inhibitor, versus a control. After incubation, cells are mildly lysed, miRISC complexes immunoprecipitated using antibodies against Ago2 or other members of miRISC and captured mRNA is isolated and quantified by microarray analysis or RNA-Seq. Directly bound targets of overexpressed miRNAs will thus be more abundantly detected (versus control), while the reverse is the case for targets of inhibited miRNAs. These high throughput methods are known as RIP-ChIP (Ribonucleoprotein Immunoprecipitation followed by microarray chip analysis) or RIP-Seq (Ribonucleoprotein Immunoprecipitation followed by High-throughput sequencing) [73,74]. Karginov and colleagues used microarrays to identify mRNA co-immunoprecipitated with c-myc-tagged-Ago2 that was stably transfected in 293S cells in combination with miR-124a mimics, detecting several targets that were affected by this miRNA only at the translational level [75]. Artifacts introduced by the use of epitope-tagged Agos (e.g., the possibility of unspecific association with RNA) can be eliminated by the precipitation of endogenous components of RISC. As an example, Tan and co-workers simultaneously inhibited four miRNAs (miR-17/20/93/106) in two different cell lines and performed immunoprecipitation of endogenous Ago2. The Ago2-associated (IP) transcripts were differentially detected by microarray as well as the transcripts present in total RNA fractions (T) of the extracts used for IP [76]. They identified more than two thousand mRNAs depleted in the IP fraction versus the total RNA (comparing IP/T ratio), and identified more than 100 candidates with a high enrichment in seed target sites in their 3' UTRs, supporting the suitability of this method to detect miRNA targets. In addition, they validated by luciferase assays all the targets assayed (nine that contained at least a 6-mer seed site for miR-17 from the list of top regulated genes), while the two most potent targets assayed without a 6-mer site in the 3' UTR did not increase luciferase activity upon miR-17 inhibition. Although the presence of those transcripts in the IPs could be due to non-specific binding, the authors do not rule out the possibility that those transcripts may be targeted through the CDS. Immunoprecipitation with Ago1 has also been used to isolate targets in mammalian cells [67,77].

One obvious limitation of this procedure is that, in many cases, the use of antibodies against only one of the several Ago proteins [11] expressed in an organism is performed. Hypothetically, mRNAs and miRNAs pulled down with different Agos may not be identical, although so far a high extent of overlapping functions has been observed for the different Agos [78]. Antibodies that recognize all Agos present in the cells or other members of RISC could preferentially be used [79,80].

Although these approaches potentially identify direct miRNA targets, they do not give information about the precise binding site and they are normally combined with bioinformatic tools and experimental validations. On the other hand, these techniques require that the miRISC-mRNA binding is stable enough to survive the IP conditions and it is known that the quantities of recovered RNA are normally very low (especially when using primary cells as starting material).

In order to stabilize the RNA-protein binding, allowing the capture of more transient interactions, the most recently developed techniques include the covalent UV-mediated cross-linking of the RNA to the proteins *in situ* preceeding lysis of the cells and the immunoprecipitation step. The first group to develop this technique for the identification of miRNA binding sites was the Darnell laboratory [81] and they termed it HITS-CLIP (High-throughput sequencing of RNAs isolated by crosslinking immunoprecipitation). The same group had previously used this technique for the identification of protein-RNA interactions in living tissues in a genome-wide manner [82]. Briefly, the method consists of the use of ultraviolet radiation to crosslink RNA-protein complexes that are, theoretically, in direct contact. The immunoprecipitation of miRISC can then be performed in much more stringent conditions (possibly decreasing the non-specifically bound material recovered). In addition, the unbound RNA is digested to leave miRISC-protected RNA fragments, which are analysed by HT-Seq. These fragments correspond to miRISC binding sites, so this technique enables mapping of the interaction sites. This first study not only elegantly proved that CLIP can be successfully used for mapping of miRISC positioning along the target mRNAs, but also made an important contribution to uncovering the mechanism governing miRNA-target recognition. A high percentage (25%) of the reads were mapped to open reading frames (ORFs), although the majority corresponded to 3' UTRs and only 1% to the 5' UTR, confirming that miRNAs preferentially bind their targets through the 3' UTR but also highlighting the importance of CDS-mediated interactions. Furthermore they found that a high percentage of the mapped sites did not contain seed sequences for any of the most abundant miRNAs in the studied tissue (brain), indicative of seedless interactions. Indeed, another study from the same group proved an alternative binding mode used by miRNAs: mRNAs containing a G-bulge site after the five consecutive nucleotides in positions 2–6 comprised >15% of all Ago-miR-124-mRNA interactions in mouse brain, and more than 75% of the non-seed detected sites [40]. The authors also found that these sites are present both in the 3' UTR and in the coding region of the transcripts and the degree of miRNA-mediated repression is similar to that observed for canonical seed binding sites.

To our knowledge, this approach has only been used so far to identify genome-wide miRNA-RISC-mRNA ternary complexes from a cell line or tissue but, its combination with over-expression/inhibition of a given miRNA could potentially be used to determine the targets of that specific miRNA.

HITS-CLIP allows mapping of miRISC binding sites with a resolution of ~50 nucleotides [81], while a modification of this method, PAR-CLIP (Photoactivatable-Ribonucleoside-Enhanced Crosslinking and Immunoprecipitation) allows identification of the precise location of the crosslink [16]. This method uses 365 nm-UV light to induce efficient cross-linking of RNA-binding proteins with photoreactive ribonucleoside analogs (such as 4-thiouridine) that are incorporated into nascent mRNAs by living cells. When this is used in combination with Ago IP, miRNA-targets are isolated, fragmented and identified by HT-Seq. When the RNA contains 4-thiouridine, the crosslinking causes thymidine to cytidine transitions, which are detected as mutations during the deep-sequencing making it possible to separate them from the background and giving a much more accurate mapping [83]. Also, the 365 nm UV crosslinking proteins-photoreactive ribonucleoside analogs are more efficient than the 245 nm protein-RNA, thus improving the precipitated RNA yields. Similarly to Chi and colleagues, Hafner and co-workers found abundant miRNA binding sites in coding regions.

These approaches may be useful not only to precisely map a direct interaction between a given miRNA and its targets, but also to detect those targets only affected at the level of translation or the interactions mediated by atypical binding sites. One disadvantage is that both PAR-CLIP and HITS-CLIP can present a selection bias for strong RNA-protein interactions, and highly expressed transcripts are generally over-represented [84]. In addition, these methods are technically extremely challenging, requiring the use of highly advanced technology not currently widely available and a very complex post-experimental (bioinformatics) analysis. Furthermore, the long multistep protocols normally require a very large amount of initial material, which is not possible in many cases, e.g., when using primary cells, and there may be potential difficulties in the combination of these approaches with the use of miRNA mimics/inhibitors. It is not surprising, then that only a few research groups are currently applying these techniques, although further optimization of the protocols may make them more accessible to other groups, which will in turn help with the validation of the technique and determination of its full applicability.

## 5. Experimental Validation of miRNA Targets

No matter how a miRNA-target interaction has been studied (bioinformatically or experimentally), it is essential to validate its functionality in the biological model of interest.

Since an inverse relationship between the levels of expression of a miRNA and its target is anticipated, the most direct and straightforward method for validation consists in mimicking/inhibiting the miRNA of interest (as previously described, generally by transient transfection) and assessing the effect in the target gene expression. Since overexpression can lead to interactions not normally occurring in the cells, inhibition should give more physiologically relevant conclusions. Nonetheless it is not always easy to inhibit the action of a miRNA to a significant extent so overexpression is more commonly performed [85].

The analysis of the effect on target gene expression should be preferentially done at the protein level (normally by Western blotting, ELISA, immunostaining, *etc*.), although due to convenience and reduced costs researchers often analyse changes in target mRNA levels instead. In that instance (as previously stated) an effect will only be observed if the miRNA affects the stability of the target, which does occur in many cases [34]. Messenger RNA changes are normally quantified by RT-qPCR, which requires low amounts of starting material and is quick and relatively cheap. Alternatively, Northern blotting can be useful, although time-consuming, especially in those cases where different isoforms of the target are expressed.

Finally, reporter assays are universally used to confirm a direct regulation of gene expression by a miRNA [21]. Generally the 3' UTR of the transcript of interest is cloned downstream of a luciferase ORF. When this vector is transfected into cells expressing the targeting miRNA, luciferase activity should be lower than for the empty vector. Inhibition of the miRNA action by co-transfection of antagonists should then result in the recovery of the luciferase expression levels, confirming that the repression is specifically mediated by that miRNA. Nevertheless, in many cases, transcription from the promoter contained in the vector is too strong and the endogenous miRNA is not able to mediate a detectable repression. In such cases, co-overexpression of the miRNA is widely used. To corroborate the direct interaction, mutation of the anticipated miRNA-binding site/s is introduced in the reporter. This mutation is expected to eliminate (or at least reduce) any miRNA-mediated effect.

Where the miRNA-target interaction is not mediated by the 3' UTR, but rather through the coding region, validation using reporter experiments can also be used, and the predicted binding site, together with the flanking sequences, may be cloned downstream of the luciferase coding region. Generally, sites in the coding region have a weaker effect on gene expression, thus their validation is more challenging.

## 6. Conclusions

Since discovered in 1993 [86], miRNAs have captured the attention of the scientific community. Nevertheless their specific functions in cells have barely begun to be unravelled. The rapid development of bioinformatics tools and HT-Seq techniques during recent years have led both to the discovery of many new miRNA classes in a multitude of different organisms and to the generation of a huge amount of data indicating their possible targets and mechanism(s) of action. Nevertheless only a small proportion of miRNA-target interactions have been functionally validated. Although each method presented here has important limitations, combining them wisely can lead to the identification of many biologically relevant targets which is essential for understanding miRNA function and for tapping their undoubted therapeutic potential.
